# Expression of pathogenesis-related proteins in potato brown rot plants confers resistance to filamentous pathogens under field trials

**DOI:** 10.1038/s41598-025-07445-0

**Published:** 2025-07-04

**Authors:** Hanaa S. Omar, Mohamed H. Hagag, Dina El-Khishin, Mona Hashem

**Affiliations:** 1 Genetics, Faculty of Agriculture, Cairo University, P.O. Box 12613, Giza, Egypt; 2 Agricultural Genetic Engineering Research Institute, Agricultural Research Centre, 9 Gama St., P.O. Box 12611, Giza, Egypt; 3 Agricultural Genetic Engineering Research Institute (AGERl), 9 El-Gamaa St., Box 12611, Giza, Egypt

**Keywords:** *R. solanacearum*, Disease control, Defense genes, DGGE gel, Biological control, Biotechnology, Microbiology, Molecular biology, Diseases

## Abstract

The present investigation aims to use innovative metagenomics technologies and denaturing gradient gel electrophoresis (DGGE) to compare the microbial communities of conductive and suppressive soils in connection to brown rot disease in the entire value of Egyptian potato imports. Besides evolution, the bioefficacy of two biocontrol agents, either alone or in consortium, on plant growth promotion and activation of defense responses in potato against the brown rot diseases. The soil status of collecting soil from seven different locations in different governorates was evaluated for tolerance to the artificial inoculation of the pathogen agent of *R. solanacearum* on potato plants. The DGGE uses 16 srRNA primers that were used to compare two extreme patterns for identifying the microbial population detected in environmental samples. Different bands were extracted from the DGGE gel and sequenced. The sequencing data results revealed that the biocontrol agent factor has a 100% gene bank similarity and belongs to the *Pseudomonas* species. The *Pseudomonas* nucleotide sequences isolates were deposited in the Gene Bank under Accession Nos. PP930812, PQ466864, and PQ470140. The findings showed that the soil from the governorates of Kerdasa exhibited a less tolerant treatment, with an estimated disease severity of 78.33%. On the other hand, ElBeheira Kom Hamada soil showed significant tolerance, with an estimated disease severity of 11.67. According to the data of gene expression analysis, both treated plants with the biocontrol agents showed a significant increase in *PR-1*,* PR-2* and *PR-Q* gene expression, which accelerated the defense response and reduced brown rot disease. The evaluated potato genotype results proved to have the potential for brown rot disease resistance and higher yield production. The findings of this study recommended that biopriming with a microbial consortium enhance potato growth, productivity, and induction of defense responses against S. tuberosum through the induction of systemic resistance via expression of *PR* pathogenic-related protein network. The present investigation offers significant perspectives that warrant further exploration in subsequent studies to address the traceability requirements of the worldwide economics of disease control for potato plants.

## Introduction

The potato (*Solanum tuberosum*) is the most important worldwide crop; Egypt ranked 10th among the top potato producers worldwide^1^. One of Egypt’s most significant crops in terms of food manufacturing and exports is the potato. On a global scale, Egypt ranks sixth in exports and fourteen in manufacturing. One of the primary fundamental principles of Egypt’s agricultural development policy is the growth of agricultural exports; according to ITC data, in 2018, the value of Egyptian agricultural exports accounted for 16% of total export value, while the value of potato exports accounted for 4.2%. In addition to being an essential food item and a grain substitute, potatoes also contribute significantly to national agricultural income and provide access to foreign cash to support and enhance Economic growth and the return on Egyptian agricultural exports^2^.

The potato crop is one of the most important vegetable crops in Egypt because of its productive and exordial position of its position; as the planted area of potatoes crop in the Republic is approximately 19.29% represents about 409.535 thousand acres of the total vegetable area, amounting to about 2,113 million acres, and the total production of Egyptian potato crop is approximately 4.61 million tonnes, representing approximately 21.15% of the total production of the Egyptian Greens, amounting to about 21.80 million tones. the value of exports of Egyptian potatoes represents 1.884 million pounds which is about 8.75% of Egypt’s total value of agricultural exports, amounting to approximately 21.070 billion pounds (representing approximately 10.8%) of the total Egyptian exports amounting to approximately 195. 276 billion pounds (current prices) in 2014^3^.

However, Egyptian potato exports are not growing at the desired rates despite the growing demand for agricultural products globally. This is especially true given the global interest in health and environmental requirements, the fierce competition Egyptian potatoes face in foreign markets, particularly the EU market, and the rejection of Egyptian potatoes by many foreign markets due to their non-compliance with these markets’ standards and brown rot, which results in low potato export values and a negative impact on both the agricultural trade balance and the country’s economy. The most significant finding relates to the summer crop of potatoes, the yield of which increased on average during the period at a rate of about 241.37% of the cultivated area, 11.9% of productivity Alfdanah, and 281.28% of kidney production; However, the average growth rate of the potato crop’s total lugs over the same time period was around 326.08% of the cultivated area, 11.63% of productivity (Alfdanah 0.269), and 61% of kidney output^4^.

In this respect, brown rot is one of the most famous diseases that cause problems in Egypt. According to the A2 European Plant Protection Organization (EPPO) during exportation, potato bacterial wilt disease, which is caused by *Ralstonia solanacearum*^5^ race 3 biovar 2 phylotype II sequevar I^6^, is one of the significant quarantines and soil-borne plant diseases. The European Union (EU) quarantine restrictions on potato brown rot, which accounted for roughly 70–90% of Egyptian potato exports, caused the total value of Egyptian potato exports to drop from a peak value of US$ 102.12 million in 1995 to US$ 7.7 million in 2000^7^. In addition, according to FAO Stat^3^, Egypt raised its usage of fungicides from 3170 tons of active ingredients in 2009 to 5080 tons in 2023, which resulted in losses for the economy and the environment.

Egyptian producers and exporters of potatoes are being forced to discover alternatives to traditional disease control measures due to the EU market’s expectations for safe food and ecologically friendly agricultural products^8^. In this respect, the search for potent and selective integrated pest management (IPM) methods has received particular attention because of the increase in antifungal resistance, which has become one of the greatest challenges for global health, food security, and development. In particular, in the agricultural field. Integrated pest management (IPM) is an important approach in sustainable agricultural development that has a role in reducing crop losses, increasing productivity, and minimizing contamination and health hazards^9^. Biological control was one of the techniques used in an integrated pest management (IPM) program^10^.

Biocontrol agents work various mechanisms to protect plants against pathogenic invasion. Using unique or a combination of processes, they may interact with the pathogen directly or indirectly to decrease plant disease^11^. Biological control agents are capable of the growth and the activity of bacterial phytopathogens in two main ways, production of anti-microbial substances and competition for space and nutrients at specific sites on the plant surface (site of competition). Anti-microbial compounds are of three main types; antibiotics, bacteriocins and siderophores. These are distinguished in terms of their chemical nature, anti-microbial activity and means of detection during in vitro culture^12^. Research on fungal strains as biocontrol agents for plant disease control has also received considerable attention. These fungi-based biocontrol agents display a significant antagonistic activity against a variation of soil and airborne plant pathogens, creation them potential biopesticides for field or greenhouse experiments^13^.It is important to determine the microbial community that live in relation with potato plants (ex. Symbiosis, parasitism, antagonism) and have a potential suppress against potato diseases^11^.

Recently, there is distinctly a partial amount of information known about the notable usage of a suitable microbial community as antagonists for the suppression of early disease in potatoes and plant growth development activities. Antagonising the survival, mobility and pathogenesis of disease causal agents were identified as suppressive soils through soil system. In this respect, microorganisms can effect on the potato plant productivity by many methods’ symbiosis with legumes plants where it can fix aeriform nitrogen in soil that increases the soil fertility. Also, *mycorrhiza* facilitate phosphorus uptake to plants. Some of microorganism called plant growth promoting *rhizobacteria* enhancing plant growth and promote the plant induce resistant or protect the plant against pathogens through the production of some metabolites such as antibiotics^11^.

According to the A2 European plant protection organization (EPPO) during exportation, potato bacterial wilt disease is one of the major quarantines for soilborne plant diseases caused by *R. solanacearum*. Microbiome of suppressive soils has always been useful tool for designing environmentally friendly against soil-borne diseases management strategy. According to a fairly recent large-scale microbiome study conducted across the worldwide regions, the most prevalent genera in the rhizosphere are Streptomyces and Lentzea, whereas *Bradyrhizobium*,* Solanacearum*,* Sphingobium*, and *Microvirga* are the primary members of the potato core microbiome^14^. The *Pseudomonadota* exhibited the largest relative abundances in the *rhizosphere*, with *Streptomyces* and *Lentzea* being the most prevalent genera. The so-called “hub microorganisms” that impact community structure through robust biotic interactions with the host and other microbial species constitute a portion of the core microbiome^14^.

Integrating advanced omics and metagenomic methods could afford a deeper understanding of the interactions between favorable microbes and pathogens. Studying the genomic, transcriptomic, and proteomic profiles of microbes, pathogens, and plants during biocontrol interactions could reveal vital mechanisms and factors inducing disease suppression^15^. It is expected that by using biotechnological approaches, researchers will be able to develop potential BCAs for efficient control of multiple crop diseases Denaturing gradient gel electrophoresis (DGGE) is a commonly used molecular technique for rapid fingerprint analysis of microbial community composition, diversity, and dynamics. The method is rapid and affordable, allowing multiple samples to be processed simultaneously. Moreover, The 16 s rRNA is a powerful tool used as molecular marker to define microbial ecology^14^. PR proteins comprise a group of inducible and functionally diverse proteins that accumulate in response to pathogen attack. These proteins have been implicated in active defense, potentially restricting pathogen development and spread^16^.

Therefore, the first objective of the present investigation was to compare the microbial communities of conductive and suppressive soils in relation to brown rot disease in the entire value of Egyptian potato imports using innovative metagenomics technologies and denaturing gradient gel electrophoresis (DGGE). Secondly, to assess how well two biocontrol agents *(Pseudomonas* spp.) promote plant growth and activate defense mechanisms in potatoes against brown rot diseases. Finally, to evaluate the gene expression analysis of pathogenic-related proteins (*PR1*,* PR3* and *PQ*) in treated potatoes with a biocontrol agent to accelerate the defense response and increase potato yield production.

## Materials and methods

### Isolation and identification of the causal pathogen of potato brown rot and wilt disease

Samples of diseased potato plants displaying brown rot and wilt symptoms were obtained during the 2022/2023 growing season from different localities. Soil, weed and potato tubers samples were collected from epidemic region AL-Monufia, Al Beheira, BeniSueif, Giza and El- Gharbia governorates to isolate the pathogen *Ralstonia solanacearum*. Soil samples were taken from 30 cm depth, by using a sampling auger that represent the major area for potato production, and usually follow different cultural practices fertilization and irrigation regimes. Ten grams of soil samples were suspended in 90 ml of sterilized phosphate buffer (0.01 M) (pH 7.0) and were shaken for 1–2 h at 100 rpm at 15 °C. Roots with crowns of potato plants or weeds were washed directly with tap water and surface sterilized by alcohol (70%). These parts were macerated in 1 ml sterile phosphate buffer 0.1 M (pH 7.0) for 5–10 min. Potato tubers showing internal symptoms were washed in running tap water and surface sterilized by alcohol (70%). Cores of 5–10 mm in diameter and 5 mm thick, containing the main vascular were macerated in 1.0 ml sterile phosphate buffer 0.1 M (pH 7.0) for 5–10 min. 100µL per samples were spread onto the semi selective medium of South Africa (SMSA medium) with three replicates and were incubated at 28 °C for 3 days^17^.

### Physiological test of the causal pathogen of potato brown rot and wilt disease

The bacterial isolate which were pathogenic to potato plants, and caused brown rot and wilt disease, were determined according to their morphological, cultural mentioned by^18^ and^19^.

### Pathogenicity tests

Pathogenicity tests of the bacterial isolate were recognized on potato (*Berema cultivar*) plants in the open greenhouse during the 2022/2023 growing season at the quarantine greenhouse facilities at PBRP, Ministry of Agriculture, Dokki, and Giza, Egypt. Virulent isolate of the pathogenic bacteria were grown on nutrient agar and King’s B medium at 28 °C for 48 h. Bacterial growth were suspended in sterilized water and were adjusted according to its optical density at A_600_nm = 0.3 to give 4 × 10^7^ colony forming units (cfu)/ml according to Michel and Mew^20^. Soils inoculations were carried out by adding 250 ml of the previous bacterial suspension to the soil treatments. One of germinated certified seed potato tubers were sown for each pot containing infested soil. The three treatments were inoculated potato plants, non-inculcated potato plants and inoculated soil without potato plants with control with each treatment. Disease severity was recorded for each plant at 6 and 8 weeks after planting. The soil that shows the highest suppressive potential and the soil with least suppressive potential were selected for determined the microbial group that cause the suppressive effect. The following formula was used to measure disease severity %: DS %= [d/d (max × n)] × 100 Where, d = the disease rating on each plant; d max = the maximum disease rating possible; n = the total number of plants examined in each replicate. Also, the virulence of the ten isolates tested was scored as follows; less than 2 were considered low, between 3 and 4 were moderate (M), and more than 4 were considered high (H). Later, the pathogen (*R. solanacearum*) was recovered from wilted plants inoculated with each of the isolates.

### Molecular analysis

#### DNA extraction and Met genomic DGGE electrophoresis method

The extraction for soil samples by CTAB DNA extraction method were followed according to van Elsas^21^. Briefly, extraction buffer components were (100 mM Tris–HCl, 100 mM Na-EDTA, 100 mM Na3PO4, 1.5 M NaCl, 1% acetyl trim ethyl ammonium bromide [CTAB], 1 mg/ml proteinase-K, 250 mg/L lysozyme, pH 8.0). Phenol/chloroform was used to get rid of protein contaminants, and DNA were precipitated by isopropanol and washed with Ethanol. Then, the DNA was quantified. Quiagen DNA purification kit was used for DNA purification. Thermo fisher Nano drop was used for DNA purity verification.

In this investigation, the microbial biodiversity in soil and rhizosphere was determined for four samples using the Decode system (Bio-Rad Laboratories, Hercules, CA, USA). The DGGE gels were prepared as described by Farag^22^. Briefly, The DGGE gels were prepared using Decode system, Universal Mutation Detection system BioRad (model 475). The vertical denaturing gradient, for 16 S Eubacteria, of 45–60% (100% denaturant is defined as 7 M urea plus 40% formamide; the gel was topped with a 8% acrylamide stack without denaturing. Decode template, prepared with Gel bond PAG film (Amersham Pharmacia Biotech AG, Uppsala Sweden) to one side, using Electrophoresis was performed in 0.5×TAE buffer for 16 h at 100 v at a constant temperature of 60 C. Gels was stained with Bio-Rad’s Silver Stain (Biorad Laboratories, Hercules, CA, USA) according to the manufacturer’s protocol. Gels were dried for 2 days at room temp. The gels were scanned using a resolution of 600 dots per inch. Scanned gels were analyzed with Phoenix 1D (Nonlinear Dynamics Ltd., Newcastle upon Tyne, UK).

#### Sequencing methods

For 16 S rDNA sequencing, DNA templates were detected for PCR amplification. DNA coding for 16 S rRNA regions was amplified by PCR with Taq Polymerase as described by Nguyen^21^.One different band was picked out and extracted by using qiagen extraction kit from poly acrylamide gel. The DNA re extracted from gel was purified and amplified by using 16 s primers. The V6 to V8 region of the 16 S rRNA gene was amplified from the extracted DNA using the primers 968 f and 1401 r as described in Elhalag^22^ (Table 1). The amplified PCR products were purified using Pure Link TM quick gel extraction kit (Invitrogen, Life Technologies, and Löhne, Germany). Twenty ng from each purified PCR product was added to 20 µl PCR and amplified according to the diagnostic procedure by ABI Prism^®^ Big Dye^®^ Terminator v3.1 Cycle Sequencing Kits (Applied Biosystems, Foster City, CA, USA). The sequencing process was conducted at the Agriculture research center laboratories (Giza, Egypt) using an 8-capillary Genetic Analyzer (Applied Bio system). The partial 16 S rRNA gene sequences (containing a sequence between U968-f and U1401-r) were compared with the sequences of the Gene Bank DNA database by using the BLASTN algorithm (http://blast.ncbi.nlm.nih.gov/Blast.cgi), and nucleotide blast was selected. Mega software was used to refine forward and reverse DNA sequences.

**Table 1:** Primer pairs used for quantitative real-time polymerase chain reaction (RT-qPCR) analysis to characterize gene expression used for quantitative real-time polymerase analysis.

Gene	Description	Gene sequence
PR1a	Pathogenesis-related protein class1	F. 5-CTTGTCTCTACACTTCTC-3 R. 5-GTATGGACTTTCGCCTCT-3
PR2a	Pathogenesis related proteins class2	F. 5-GCAACATATTCAGGGATC-3 R.5-ATTGAAATTGAGTTGATA-3
PRQa	β−1-glucanase.	F. 5-CCAGAGTGACAGATATTA-3 R. 5-GCCCTGGCCGAAGTTCCT-3
Coxa	Housekeeping.	F. 5-CGT CGCATT CCA GAT TAT CAA-3 R. 5-CAA CTACGG ATA TAT AAG AGC CAA AAC TG-3
16 S rDNA	16 s rRNA gene	F −5GAGTTT GAT CCT GGC TCA G-3’ R. 5’-GTT ACC TTG TTA CGA CTT-3

### Isolation and identification of the biological strains

For the isolation of biological strains from Seven potato rhizosphere soil samples were collected from (El-Monufia-Ashmon, El-Gharbia-Kafr El-Zayat, El-Monufia-El-Bagour, Giza-Kerdasa, El-Beheira-Kom Hamada (1), El-Beheira-Kom Hamada (2), and El-Beheira-El-Tawfequea) (Table 2). All experiments were carried out under artificial inoculation conditions in a greenhouse. All greenhouse experiments were performed with one of the previous ten isolates, which were isolated from tubers and showed more aggressiveness on potato plants. A subsample of 10 g was obtained from the soil samples from each location, placed in 250 ml Erlenmeyer flasks with 100 ml sterilized distilled water (DW), and mixed for 10 min with a magnetic shaker. From this suspension, a dilution series up to 6–10 was prepared. When the bacterial colony appeared on the medium, representative isolates were picked for this study. Pure cultures of bio control agent strains were determined using the morphological and physiological characteristics according to the methods of Lelliott and Stead^23^.When the bacterial colony appeared on the medium, representative isolates were picked for this study. Pure cultures of bio control agent strains were determined using the morphological and physiological characteristics.

**Table 2:** Isolation of pathogenic bacteria in soil, tubers and weeds plants samples collected from potato field, at different governorates, during growing season 2016–2017.

Governorate	Source of sample	Total number of samples	Positive sample	
			No.	(%)
Al-Beheira	Soil	23	16	69.6
	Tuber	86	73	84.9
	Weed	14	8	57.1
Al-Menofia	Soil	18	12	66.7
	Tuber	64	51	79.7
	Weed	11	6	54.5
Beni-Suief	Soil	10	6	60.0
	Tuber	32	23	71.9
	Weed	7	3	42.9
Total samples	Soil	51	34	66.7
	Tuber	182	147	80.8
	Weed	32	17	53.1

### In-vitro evaluation of potential antagonists

Two antagonists namely *Pseudomonas putida* was evaluated against the bacterial wilt pathogen in vitro. The experimental designs were complete randomized design (CRD) with four replications. Cross culture method and filter paper disk method were used in first experiment and second experiment, respectively. PDA medium was used in both experiments in order to favor the growth of *R. solanacearum* and the potential antagonists. Of these antagonists, two most effective antagonists were selected based on the degree of inhibition of pathogen and growth rate of antagonist for in-vitro evaluation studies.

### In-vivo evaluation of potential bio-control agents

The two selected potential antagonists (*Pseudomonas putida* was evaluated in vivo against *R. solanacearumin* the greenhouse using susceptible potato variety marmand, the experimental designs were complete randomized design (CRD) with five Replications (3seedlind/plot). The temperature and relative humidity of the greenhouse were set at 30ºC and 80% respectively in order to favor the disease development. In pot experiment, the antagonists were introduced one week before the pathogen inoculation. Two selected antagonists were applied to 21-days-old potato seedlings growing in separate pots filled with sterilized soils. Antagonists were applied regularly up to 6 times at one-week interval. To apply antagonists, 15 mL of suspension at a concentration of 109 cfu/mL of each of the six selected antagonists were used. After 40 days of planting percentage of disease reduction was evaluated from each treatment, fresh and dry weight of shoot, fresh and dry weight of root, 10 plants were used for evaluating potato plants yield.

### Quantitative real‑time PCR (qRT‑PCR)

Total RNA was extracted from leaf potato plants collected from the Treated plants with bio control agent during artificially infected with pathogenic using the RNeasy^®^ Plant Mini Kit (Qiagen, Hilden, Germany) according to the manufacturer instructions. A total of 1 to 2 µg of DNase I treated RNA was reverse transcribed using Prime-Script First Stand cDNA Synthesis Kit (thermo kit). C DNA was analyzed by quantitative real-time PCR using SYBR kit (Takara) in Thermal Cycler Bio-Rad Real Time System II (- (Bio-Rad, California, USA). Primers specific for pathogenesis related genes (*PR1*,* PR3* and *PQ*) expressed were designed using primer blast (https://www.ncbi.nlm.nih.gov/tools/primer-blast/) and primer3 software as shown in Table 1. Those selected genes covered the main group of defense genes including; pathogenesis related genes (*PR1*,* PR3* and *PQ*) Primers were used in RT-qPCR analysis to amplify fragments of ~ 80–200 bp in length using the Takara SYBR^®^ Premix Ex Taq™ in 25 µl reactions containing 60 ng template cDNA and 5 µl of 1 µmol/l of each oligonucleotide, 12.5 µl SYBR premix ExTag. Amplifications were performed by initial denaturation at 95 °C for 120 s followed by 40 cycles at 95 °C for 15 s and at 60 °C for 30 s. Melting curves were analyzed for each data point to check the specificity of the PCR product. For different gene expression results, the delta-delta Ct was calculated. Average CT values were calculated from the triplicate experiment conducted for each gene as the CT value detected by subtracting the average CT value of genes from the CT value of Coxa gene. Actin1 was used as housekeeping genes to normalize cDNA concentrations.

### Plant growth parameters

At 40 days after transplantation, the effects of bio control agent treatments on potato plant growth were determined by assessing the maximum plant height.

### Statistical analysis

All growth parameter characteristics will be subjected to ANOVA (tow way ANOVA with interaction post-hoc, LSD) to test for differences between these parameters and treatment types and their concentrations, using SPSS v 16. AUDPC data will be transformed by equation X=√(X+3/8). Disease index percentage data were transformed by equation P=√(X+3/8/n)+3/4 Pathogen population in soil data were transformed by equation X =(log×+10) and was subjected to ANOVA Repeated measurement with interaction post-hoc, LSD^21^.

## Results

### Isolation, identification, morphological and molecular test of the causal pathogen

#### The disease symptoms

**Fig. 1:**
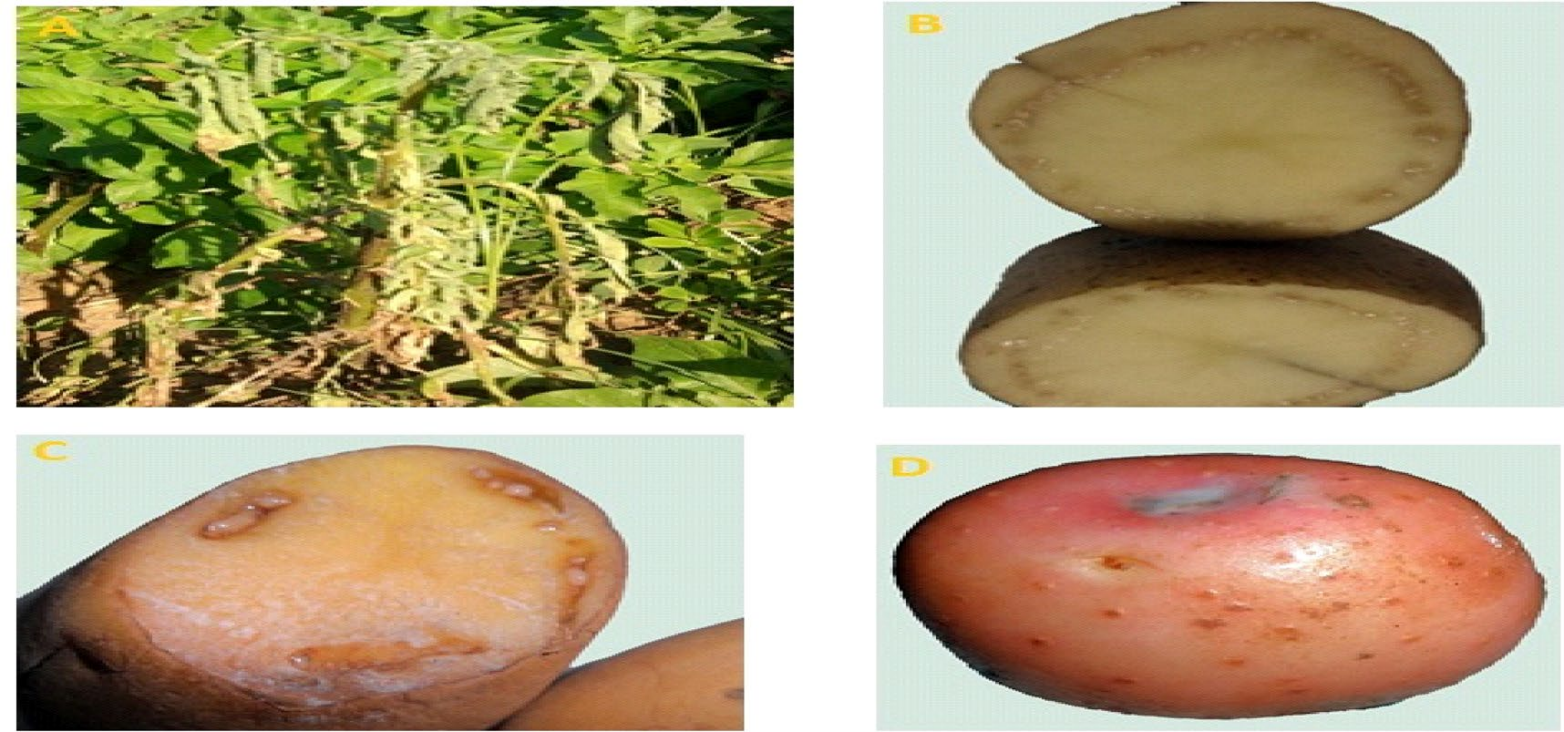
Typical symptoms of Potato bacterial wilt disease naturally infected potato plant in field (**A**) and tubers (**B**, **C** and **D**) showing brown vascular discoloration and ooze in severe infection.

Typical symptoms of bacterial wilt disease of potato were recorded under naturally infection conditions at different governorates, during 2022/2023 growing seasons to isolate the causal agent of potato bacterial wilt. The first visible symptoms are wilting of the lower leaves with rolling of the leaf margins (Fig. 1A) and infected plants fail to recover and die. A streaky brown discoloration of the stem may be observed on diseased stems above the soil surface. On tubers, in the later stage of disease development bacteria pass through the vascular tissues and will emerge from the eyes as sticky, dirty-white (Fig. 1B), often bubbly masses to which the soil readily adheres. Cutting the diseased tubers revealed a browning and necrosis of the vascular ring and immediately surrounding tissues side of the ring (Fig. 1B). A creamy fluidal exudate usually appears on the vascular ring of the cut surface (Fig. 1C and D).

### Isolation of causal agent

**Fig. 2:**
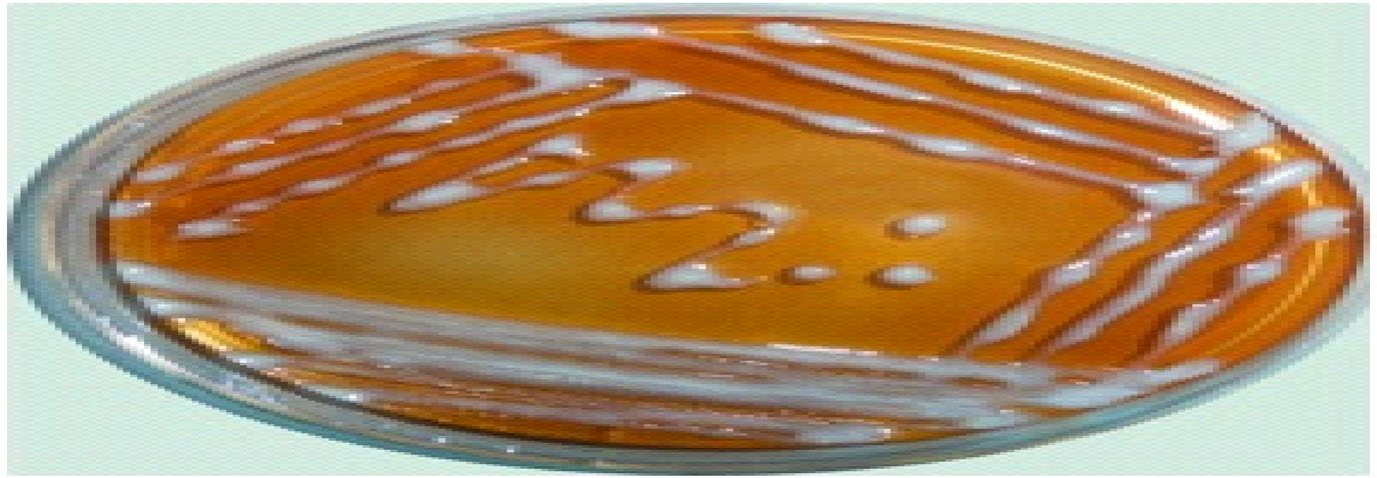
*R. solanacearum* colony morphology on kings B medium.

**Fig. 3:**
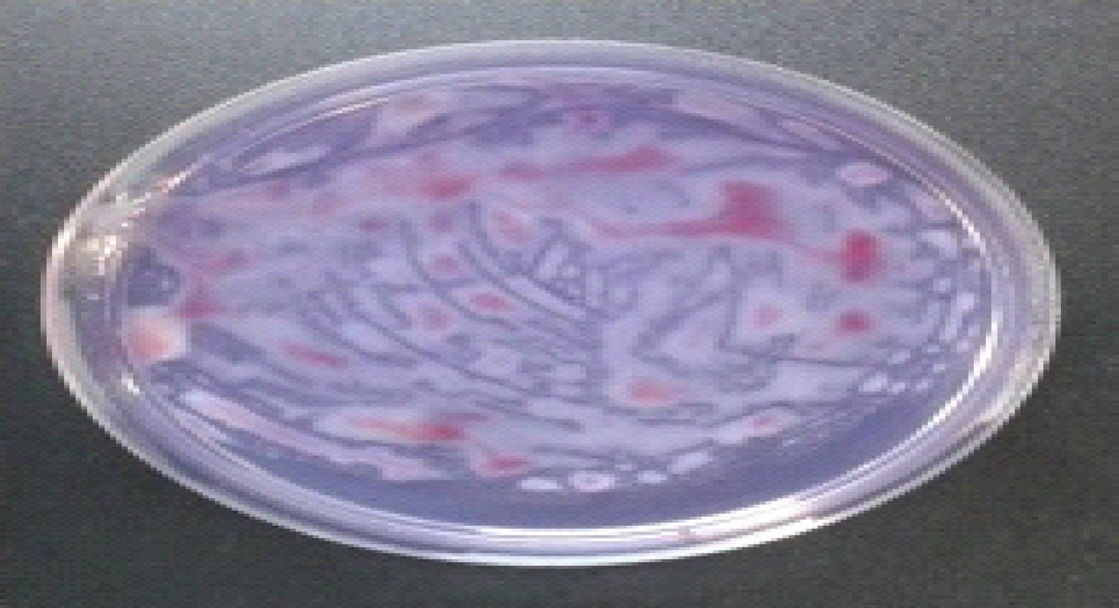
Colony morphology on SMSA semi selective medium of R. solanacearum.

Isolation of pathogen was carried out from samples of soil, tubers and weed plants on the modified SMSA medium selective for the pathogen (Figs. 2 and 3). One hundred eight nine bacterial isolates were obtained from diseased potato plants display typical symptoms of bacterial wilt collected from different counties. These samples were collected from Al-Beheria, Al-Mmenofia and Beni-Sueif governorates, during growing seasons 2016–2017 as shown in Table 2. Approximately, out of 51, 182 and 32 total samples of soil, tubers and weed plants were collected from governorates, respectively, only 34, 147 and 17 samples were positive samples for occurrence of *R. solanacearum*, respectively and recorded as isolates. Detection of *R. solanacearum* bacterium was highly recorded with tubers samples (80.8%) followed by soil samples (66.7%) and was lowest recorded with weeds samples (53.1%). Meantime, positive samples in soils, tubers and weeds were highly recorded in Al-Beheria governorate (69.6, 84.9 and 57.1%) respectively followed by Al-Menofia governorate (66.7, 79.7 and 54.5%) respectively and were lowest in Ben- Sueif governorate (60, 71.9 and 42.9%), respectively.

### Pathogenicity test

**Fig. 4:**
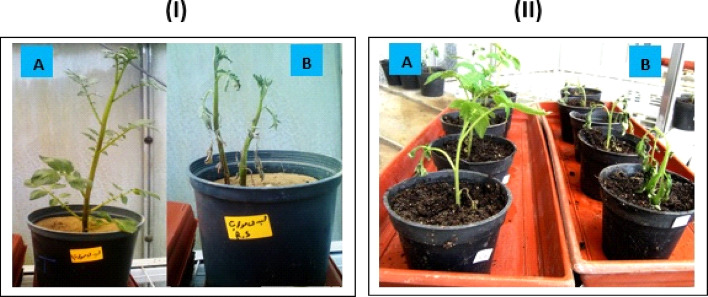
Pathogenicity test on potato (I) and tomato (II) plants, where (**A**) non-inoculated plants as the control, (**B**) inoculated plants.

The test was carried out on tomato seedlings (3- leaves) and potato plants (30 days) using different isolates which isolated previous, under artificial inoculation conditions. The symptoms were recorded after 10 days on tomato seedlings, after 30 days on potato plants and after 60 days on potato which inoculated by atypical form of the pathogen, where inoculated plants showed wilting, stunting and yellowing of foliage compared with non- inoculated plants as control treatment (Fig. 4). Results revealed in Table 2 exhibited that all studied bacterial isolates were pathogenic to potato plants and created typical symptoms of the wilting disease on infected plants. However, the bacterial isolates significantly various in their virulence to potato plants. The isolates No. (73), (51) and (147) were the highest ones, where they exhibited average 81% of wilt disease severity. While the isolates No. (3), (6) and (17) were the lowest ones and caused average 49% of wilt disease severity. The other bacterial isolates ranged from 57.1-71.9% of wilt disease severity. Depend on the data acquired; the bacterial isolates could be characterized as high virulent [No. (73), (51) and (147), moderate virulent [No, (3), (6) and (17), and low virulent were other remaining bacteria.

### Identification of the causal pathogen of potato wilt disease

#### Morphological and cultural characters

The identification of the pathogenic isolate bacteria in the present study was performed using morphological characteristics. Ten pathogenic isolates were selected according to their severe reaction in pathogenicity test and colony morphology on SMSA medium. The results revealed that all bacterial isolates were short – rod, negative staining and non-sporulation. Developed colonies on nutrient agar (NA) medium were irregularly – round, convex, smooth surface, entire margin, translucent and yellowish brown in color (Table 2). Meantime, these colonies were whitish- gray in color on King’s B (KB) medium forming brown pigments in most cases. Colonies on tetrazolium chloride (TTC) medium and semi-selective medium of South Africa (SMSA) were fluidal white with red center.

#### Serological and molecular characteristics

**Fig. 5:**
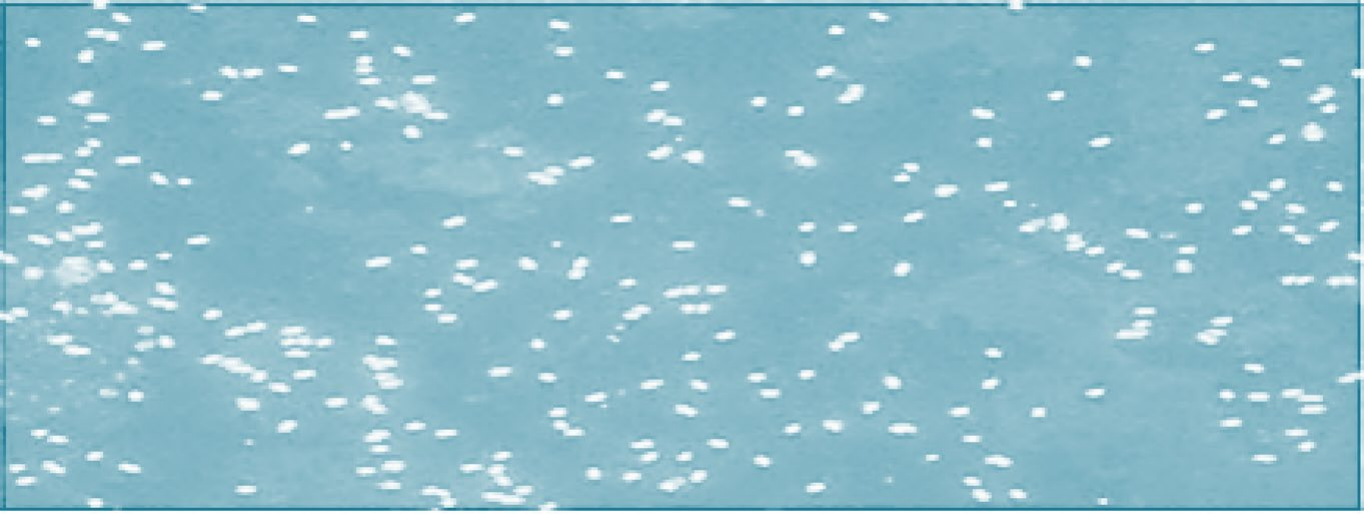
Immunofluorescent antibody staining (IFAS) test, the apple green fluorescent of *R. solanacearum* cell were appeared.

**Fig. 6:**
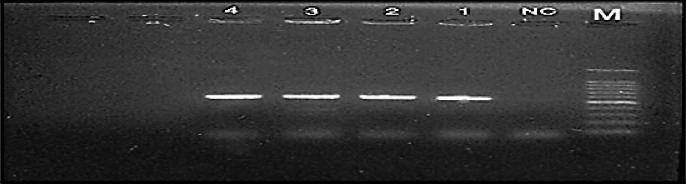
PCR gel electrophoreses of *R. solanacearum* isolate using specific primer. M: Marker, NC : Negative control, lane 1–4 R.S isolates.

The results of Identification of the tested bacterial isolates using immunoflurescent antibody staining technique (IFAS) as serological test of the pathogenic isolates gave positive result where it confirmed that these tested isolates are *R. solanacearum*. In this respect, the morphology of bacterial cells appeared as short rod shape and green fluorescent with specific fluorescent-labeled antiserum as cleared in Fig. 5. Therefore, all bacterial isolates causing wilt disease of potato were identified as *R. solanacearum*. Identification methods for the pathogenic isolates were carried out through using Immunofluorescence antibody stains (IFAS). The most five *R. solanacearum* pathogenic isolates showed fluorescent short rod shape cells and evenly stained as bright green fluorescent under immunofluorescence microscope (Fig. 5). Also, the result represent that the probe used in *Taq-man* was of the type (B2) capable to detect only biovar 2 of *R. solanacearum* bacterial wilt. Furthermore, primer and probe are specific for detection of the race 3 biovar 2 strain. Positive results were obtained in assays with all ten isolates. The curve approached from number (1) on horizontal axis which indicates to cycle number was the most virulence isolate compared to negative control. Also, the result represent that the specific primer used in PCR of the type (B2) capable to detect only biovar 2 of R. solanacearum bacterial wilt as shown in Fig. 6.

### Bio control agent identification using 16 s rRNA gene amplification

**Fig. 7:**
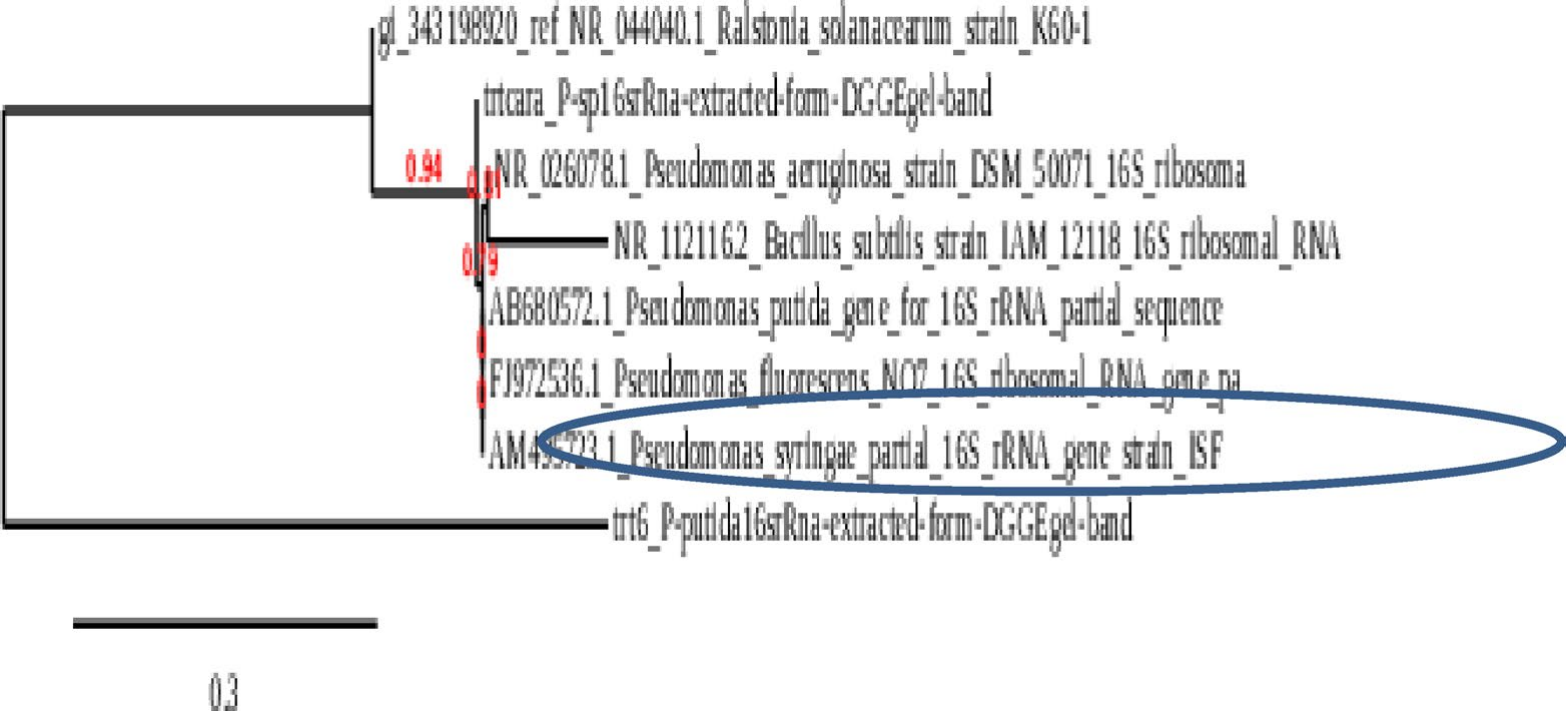
Identification of bio agent *pseudomonas putida* factor that responsible for Tolerance against R. *solanacearum* using DNA sequencing.

**Fig. 8:**
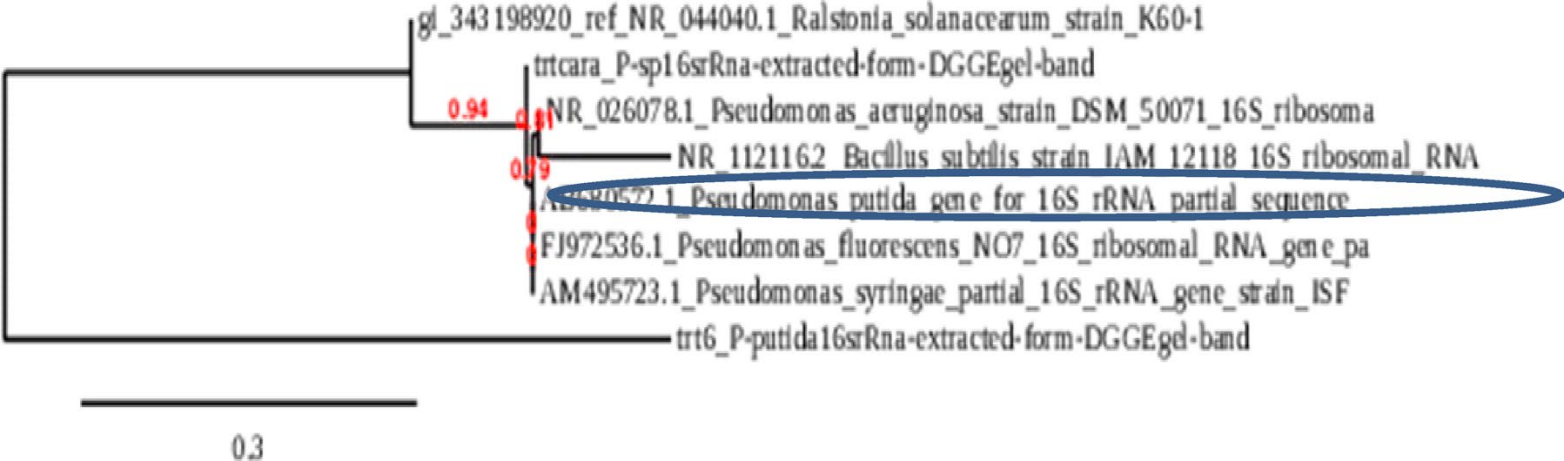
Identification of bio agent *pseudomonas florescencs* factor that responsible for Tolerance against R. solanacearum using DNA sequencing.

**Fig. 9:**
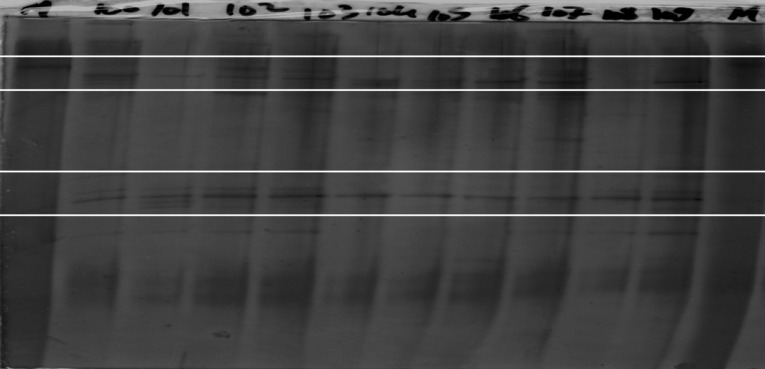
A DGGE gel was created from two extreme samples. (1) Conductive102 and (2) suppressive103 using 16srRNA primers, which show variant bands.

The molecular identification and phylogenetic analyses of the purified pseudomonas spp isolates were carried out. The PCR product of the ITS sequence was identified. The sequence analysis of the bio agent control isolate showed high similarity to the two strains of pseudomonas spp ITS sequences and it were deposited in the Gene Bank under the two accession numbers.1. In addition, the phylogenetic analysis confirmed that it had the highest similarity to the pseudomonas spp ITS sequences isolate. The results of the phylogenetic tree analysis revealed that the closeness of the genetic similarity between the studied pseudomonas spp ITS sequences isolate and others from around the world was mainly with strains of pseudomonas spp ITS sequences with different accession in the database of the Gene Bank (Fig. 2b). Therefore, pseudomonas spp was recognized as the bio control agent mediator of the pseudomonas in potato in Egypt. Using partial 16 S rDNA sequencing analysis showed that the 1 st strain belongs to the genus *Pseudomonas*; with closest similarity *to pseudomonas putida* 100% similarity have accession number (PQ466864) as presented in Fig. 7..Moreover, the 2nd strain belongs to the genus Pseudomonas, with closest similarity to *pseudomonas plecoglossicida* 99% similarity (Fig. 8). The nucleotide sequence of the P. putida and P. *plecoglossicida* isolate were deposited in Gene Bank with Accession No. PQ466864 and PQ470140, respectively. Moreover the DGGE analyses are most effective for the analysis of samples with lower relative diversity. Nonetheless, in this investigation, have successfully employed DGGE for comparisons of complex microbial communities, e.g. those found in soil samples as shown in (Fig. 9).

#### In vitro evaluation of antagonistic activity of *Pseudomonas putida* and pseudomonas florescence against *R. solanacearum*

**Fig. 10:**
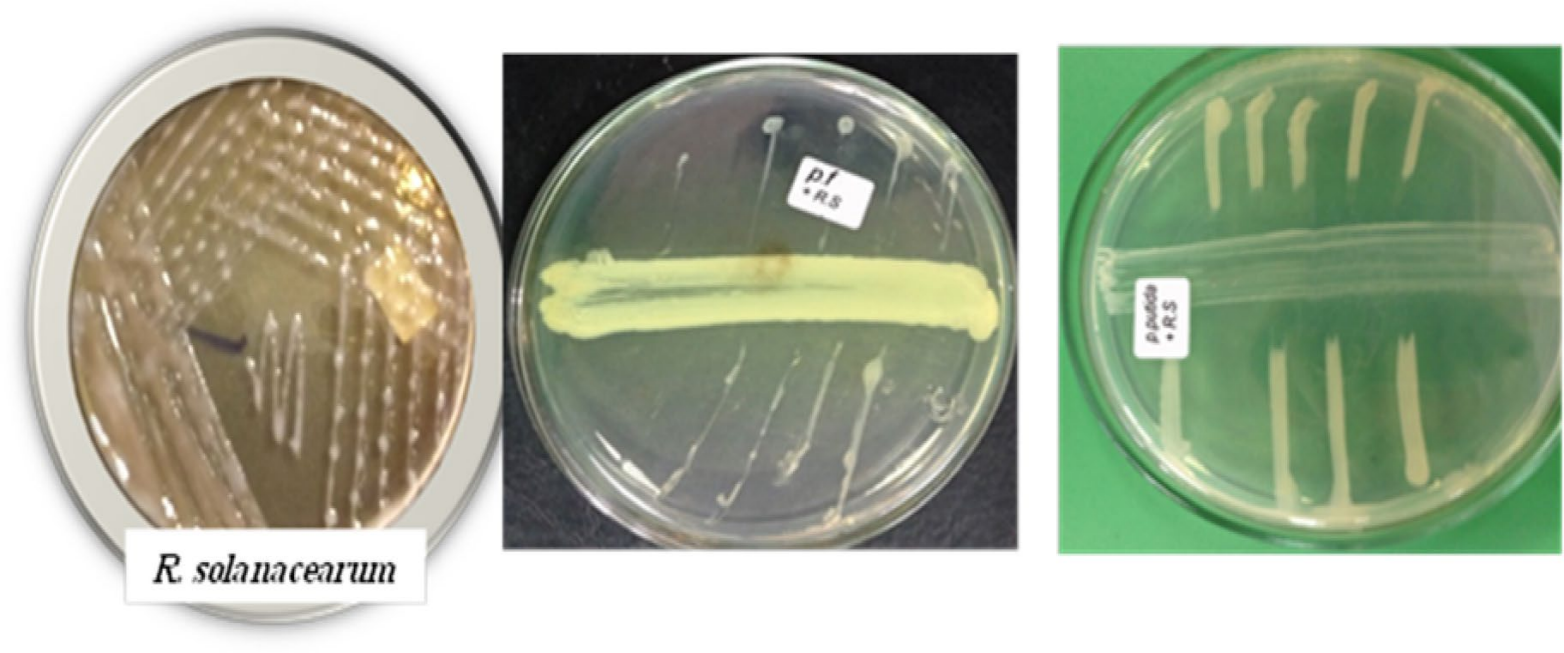
The antagonistic ability of pseudomonas spp. against R. solanacearum under in vitro conditions.

The *pseudomonas* species were evaluated to check their efficacy against R. *solanacearum* under in vitro conditions. The results of the experimental data are presented in (Fig. 10). When compared to controls, all of the tested pseudomonas species significantly inhibited the test pathogen’s mycelial growth. Among the two tested pseudomonas species (pseudomonas florescence) resulted in maximum mycelial growth inhibition by 95%. While, *pseudomonas putida* showed mycelial growth inhibition of 88.66%. A significant variation was recorded among the species of pseudomonas to form an inhibition area against *R. solanacearum*. Maximum inhibition area against *R. solanacearum* was recorded in the treatment of *pseudomonas putida* (8.7 cm2) followed by p. florescence (6.9 cm2) isolates designated was selected for this investigation. The antagonistic activity of the *p. putida* and *p. florescence* are presented in and Fig. 10. The detected radial inhibition zone for this isolates, and there were significant variations in the mean diameter of the inhibition zones (*P* < 0.05(.

### In-vivo evaluation of potential bio-control agents

The results presented indicate that all bio control agent had the ability to reduce the growth of *R. solanacearum*. The most effective reduction of disease symptoms was obtained by using *pseudomonas putida* and *pseudomonas florescence* showed the reduction of disease symptoms by using these two organisms. Soil application with *Ralstonia solanacearum* inoculation were shown various signs and symptoms on potato plants after 40 days compared with negative control, Giza – Kerdasa, El- Beheira – El-Tawfequea, El- Monufia – Ashmon, El- Monufia – El-Bagour, El- Gharbia – Kafr El-Zayat, El- Beheira – Kom Hamada (1), El- Beheira – Kom Hamada (2) as presented in Table 2 (78.33, 76.11, 63.89, 61.67, 58.89, 37.22, 11.67) respectively. Soil that collected from Giza – Kerdasa were shown the less tolerant against the bacterial wilt disease with percentage of disease severity estimated with 78.33% opposite to El- Beheira – Kom Hamada (2) that had shown high suppression against the pathogens with percentage of disease severity estimated with 11.67%. The two extreme results of disease severity of Soil of Giza – Kerdasa and El- Beheira – Kom Hamada (2) treatments had been chosen to DNA extraction, PCR-DGGE analyses of 16 S-rDNA from soil samples of the extreme treatments revealed that were clearly different band that picked up for purification then for DNA sequencing.

### Determination of *in planta* gene expression of either pathogenesis related protein genes in potato and genes encoded for proteases in the bio-control agent

**Fig. 11:**
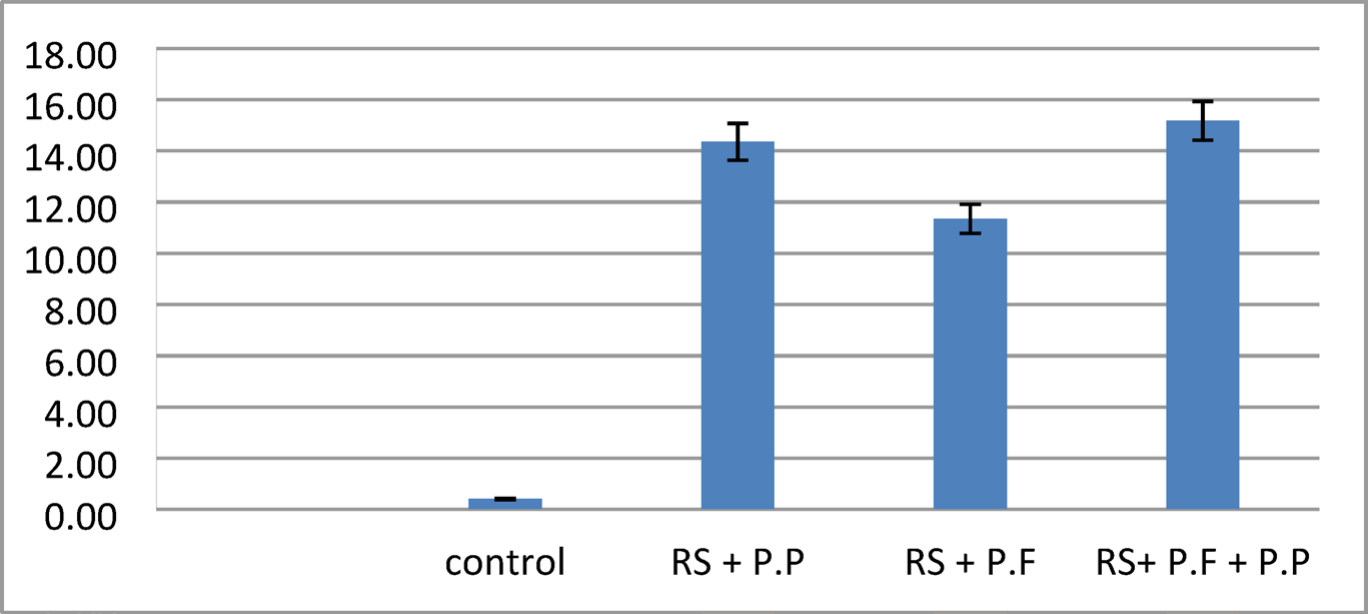
Relative expression for expression of pathogenesis related protein derived plants (PR1) of potato leaves cv. Lady Rosetta simultaneously inoculated by R. solanacearum race 3 biovar 2, in greenhouse conditions.

**Fig. 12:**
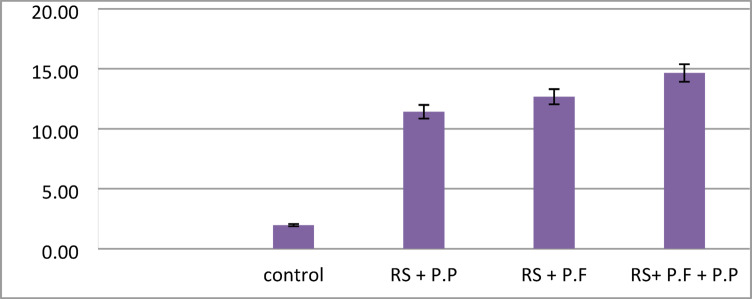
Relative expression for expression of pathogenesis related protein derived plants (PR2) of potato leaves cv. Lady Rosetta simultaneously inoculated by R. solanacearum race 3 biovar 2, in greenhouse conditions.

**Fig. 13:**
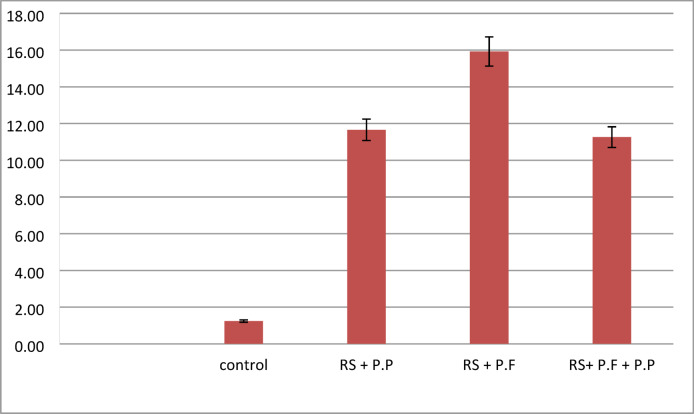
Relative expression for expression of pathogenesis related protein derived plants (PRQ) of potato leaves cv. Lady Rosetta simultaneously inoculated by R. solanacearum race 3 biovar 2, in greenhouse conditions.

For studying the gene expression of pathogenesis related protein genes in potato plant, six replicates from each treatment as one replicate for each stage were used for gene expression of specific marker genes in plant which encoded for pathogenesis related proteins i.e. *PR-1*,* PR-2* and *PR-Q.* These stages include: vegetative growth (at 30 days after potato cultivation), tuber initialization (at 45 days after planting), tuber bulking (at 65 days after planting), and maturation (at 80 days after planting). Plants respond to pathogen attacks by activating the synthesis of a diverse number of defense proteins. Corroborating this, comparative analysis of disease related genes of the bio control agent treated potato with bio control agent under pathogenic condition were carried out as an attempt to understand the indirect effects of bio control agent. The expression profile of three functional genes belonging to the three important identified genes related to biotic stress response were evaluated in order to clarify the plant response during the interaction with the bio control agent and pathogenesis related protein (*PR1 & PR3*). Analysis of the gene expression indicated that all of the identified PR genes were transcribed in potato plants after treatment by *Pseudomonas spp*. The maximum levels of the PR1 expression gene were determined in the plants treated with *Pseudomonas putida* as presented in Fig. 11; in addition, *PR2* was expressed in potato plants after treatment by Pseudomonas fluorescence Fig. 12. A strong expression of *PQ* was observed in potato plants after treatment by Pseudomonas fluorescence Fig. 13. The results found that all PR genes were upregulated in *R. solanacearum* with the bio control agent. In particular, expression of PR-1, PR-2, and PR-Q expression genes increased up to 16 folds, supporting that the PR gene family plays important roles in biotic stress response in plants. Significant fold differences for the expression of selected genes in the treated plants with bio control agent after pathogenic were observed. The expression levels for pathogenesis related genes. Relative expression for expression of pathogenesis related protein derived plants (*PR-1*,* PR-2 and PR-Q*.) of potato leaves cv. Lady Rosetta simultaneously inoculated by *R. solanacearum* race 3 biovar 2; in greenhouse conditions were increased compared to the untreated plants as presented in Figs. 11, 12 and 13. The results revealed that the transcription level of the SA-inducible genes *PR1* and *PR3* plays an important role in the defense-response in plants.

### Potato yield production

Results of the potato plants treated with bio control agent displayed that the plant treated with bio control agent pseudomonas putida had significantly highest yield under pathogenic infection pseudomonas putida, whereas the no treated plant the lowest yield pathogenic conditions. The data presented in Table 3 revealed that El- Beheira – Kom Hamada (1) treatment was shown the higher significant values of Shoot_wet_wieght Shoot_Dry_wieght and tuber weight than the other treatments. Kerdasa treatment was shown the less treatment values of Shoot_wet_wieght Shoot_Dry_wieght. Moreover the data presented in Table 4 demonstrated that No significant differences in (Shoot_wet_wieght Shoot_dry_wieght and tuber weight) between the treatment Rs + PP, Rs + Pf and Rs + PP + PF Compared with control. All treatment was shown significant suppression against RS Compared with positive control.

**Table 3:** Physiological parameter of seven treatments that show shoot wet weight, shoot dry weight and tuber weight.

Treatment	Shoot wet weight	Shoot dry weight	Tuber weight
El-Monufia–Ashmon	56.55	10.96	51.42
El-Gharbia–Kafr El-Zayat	73.3	10.76	59.48
El-Monufia–El-Bagour	60.4	11.50	49.88
Giza–Kerdasa	54.54	11.90	44.78
El-Beheira–Kom Hamada (1)	86.28	19.11	82.62
El-Beheira–Kom Hamada (2)	79.82	13.41	51.64
El-Beheira–El-Tawfequea	59.7	11.48	46.84

**Table 4:** Potato physiological parameter that treated with bio-control agent.

Treatments	Shoot_wet_wieght	Shoot_dry_wieght	Tuber weight
Pc	15.62	10.46	22.12
Nc	66.28	19.11	82.62
Rs + PP	51.88	17.84	69.37
Rs + Pf	53.91	16.41	68.84
Rs + pp + pf	54.57	13.5	57.45

## Discussion

Potatoes are an essential food crop in the globe, and their growth and yield are greatly influenced by the structure and variety of the bacterial and fungal communities that they reside together with. The soil surrounding the tubers, a particular portion of the potato plant, is referred to as the “geocaulosphere.” This sub-compartment is typically linked to the storage process and the tare soil microbiota^24^.Bacterial wilt and brown rot of potato plant caused by *Ralstonia solanacearum* is a devastating disease that can spread to temperate, tropical, and subtropical regions of the world. In many undeveloped countries as well as Egypt, it is a limiting factor for the production of potato tubers. Also, the pathogen has an extensive host range of 200 plant species in over 50 families, and the family *Solanaceae* is one of the most economically essential host plants. However, the precise host range and distribution of *R. solanacearum* based on the race and, to some extent, the biovars influence. It is a factor that restricts the production of potato tubers in Egypt and many other developing countries^24^. Additionally, two hundred plant species from over 50 families make up the pathogen’s wide host range^25^, with the *Solanaceae* family being one of the most economically important host plants. Nonetheless, the race and, to some extent, the biovars influence the precise host range and distribution of R. solanacearum^26^.In this investigation, ten bacterial isolates were gotten from wilted potato crops in Ashmon, Kafr El-Zayat, El-Bagour, Kerdasa, Kom-Hamada (1,2), El-Tawfequea). Later, they were recognized as *R. solanacearum* race 3 (biovar II) according to their morphological, cultural and molecular characteristics defined by^18^ and^19^). Also, these isolates were similar to those of race 3 (biovar II) of *R. solanacearum* designated by Mikhail^27^. The biovars of *R. solanacearum*, which are mostly depend on acidification of medium during the metabolism of sex sugars, are fit definite than their races^28^.

In Egypt, the biovar II of race 3 is usually establish^27^, which infect potato plants and foundation wilt symptoms in several regions^29^.Pathogenicity tests verified that all ten bacterial isolates of *R. solanacearum* were pathogenic, persuaded wilt symptoms typically, and desolated from infected potato crops. However, these isolates significantly diverse in their virulence and categorized as high virulent (nn), moderately virulent (nn), and low virulent (nn). Such results are in contract with those stated by^30^. In the present study, the low virulent isolate produced symptoms comparable to those produced by the moderate or high virulent isolate when getting access to the host potato plant. Numerous control procedures of bacterial wilt have been recognized. Conversely, it is still complex to control since of the wide host range and long survival period of the pathogen in the soil, particularly in deeper layers^31^. As per literature, to date, no particular control method is 100% effective. In this respect, control of bacterial wilt has been potential through the usage of a combination of diverse lines, and these methods include *Phytosanitation* and cultural practices^32^.Consequently, integrated and sustainable disease management options are required to control this highly destructive challenged disease^33^. Lately, it has developed necessary and highly relevant to discover the plant growth-stimulating *rhizobacteria* that display antagonistic activities to the pathogen and incorporate them into successful disease management as biocontrol agents. Also, a key feature of such organisms is their capability to adjust to the *rhizosphere* and to aggressively colonize the host plant roots^34^. Natural plant products are essential sources of new agrochemicals for the control of some plant diseases. In this respect, plant-derived preparations depend on plant extracts are environmentally safe alternatives and possible components of integrated disease management^35^.

The causal pathogen of potato brown rot *R. solanacearum* is categorized currently into five races affecting hosts in different plant families^36^. Race 3 biovar 2, the so- called potato race, is the dominant strain in Egypt^37^. Likewise, the results demonstrated that fluorescent short rod shape cells of pathogenic bacteria stained evenly as bright green fluorescent under the immunofluorescence antibody stain (IFAS) to approve the characterization of *R. solanacearum* in potato tissue and is considered as another achievement technique. According to Janse^38^, using an immunofluorescence antibody stain to *R. solanacearum biovar* 2 was shown to be detectable as few as104 CFU per mL in potato tissue. The sensitivity of serological methods for the detection of *R. solanacearum* in potato or in soil can be increased by applying an enrichment procedure, for example by inoculating extracts in a selective broth^39^. The present study, planned to clear up the sensibility of potato varieties to R. solanacearum was performed to screen variety Egyptian potato varieties for their sensibility to the pathogen as establishment to recover the management of potato bacterial wilt. No resistance against *R. solanacearum* is available in the studied potato cultivars in the present study, moreover the level of susceptibility diverse significantly amongst them. As all identified potato cultivars in Egypt are susceptible; breeding programs should to research of resistance in the wild solanum types that show tolerance or resistance the disease to achieve positive results^40^.

DGGE are most effective for the analysis of samples with high community diversity prevents clear fingerprint resolution or populations of interest exist at less than 1% of the total community, taxon-specific^41^ primers help to partition the community to focus on taxonomic subsets. Alternatively, novel two-dimensional polyacrylamide gel analysis of variable length PCR products from the spacer region between the 16 S rRNA and 23 S rRNA genes is also a powerful approach to comparing the taxonomic composition of a small number of highly complex communities^42^. Of relevance to bioremediation, functional gene primers may also be appropriate targets for DGGE fingerprinting to characterize subpopulations that are responsible for particular metabolic functions. Functional genes characterized by DGGE include methyl coenzyme-M reductase^43^, dissimilatory sulfite reductase, monooxygenase genes including ammonia and methane monooxygenases^44^, dioxygenase genes^45^, and nitrite and nitrous oxide reductases^46^.

The results of the study objectives that there are losses in potato production in infected plants compared to healthy ones, due to the severity of the disease and spreading the bacterial pathogen into plant tissues. *R. solanacearum* is a soil-dwelling bacterium that invades plants through roots and colonizes xylem vessels^47^. The present study was well-matched with some other studies, the potato production and yield losses lead to bacterial wilt as high as 100 per cent have been demonstrated in parts of tropical Africa^48^. In Kenya, the potato industry is threatened by bacterial wilt (BW) because soils in most production areas are infested with the wilt causing bacterium and over 50% yield losses have been indicated^49^.The farmers reported experiencing yield losses ranging from 5 to 80% due to bacterial wilt. According to some recent studies, the disease is found in all the potato growing areas of Egypt and the country is affecting 77% of potato farms which had been introduced with tuber seeds imported from Europe^50^. Potato yield losses in Uganda estimated about 30%^49^, with more severe losses being 100%. Kabeil^5^ reported that potatoes were one of the largest exported crops in Egypt. Yet, the total value of Egyptian potato exports fell from a peak value of US$ 102.12 million in 1995 to $US 7.7 million in 2000 mainly due to this organism related quarantine restrictions imposed by the European Union (EU) which used to account for about 70–90% of Egyptian potato exports and it represented a drop from approximately 419,000 metric tons to 48,500 tons.

Meanwhile, the potato varieties with the least susceptibility to the disease would be studied when implanting potatoes in fields with a history of bacterial wilt. Therefore, we must go to build up programs to discover resistant and/or tolerant potato varieties^51^. In this investigation, results indicate that the two bacterial isolates produced from naturally diseased potato plants collected from different localities confirmed to be pathogenic and capable of infection potato plants causing wilt symptoms and varied in their pathogenicity. They determined as *Ralstonia solanacearum*. The results agreement with those reported by^52^ the results demonstrated that *Ralstonia solanacearum* generated fluidal and irregular colonies with pink or light red at centers at 30^o^C after 48 h of incubation. Constructed on the results of this research, we can conclude that the all bio-control agents tested showed their ability to decrease the severity of bacterial wilt disease and increased percentage of germination. Some of these specifically belonging to *P. aeruginosa* caused substantial increase in plant growth and yield. They would fall under the category of plant growth promoting bacteria (PGPB). Pseudomonads also play a role in growth promotion by production of plant hormones and other growth promoting substances. In vitro conditions, results clearly confirm that plants treated with pseudomonas putida and pseudomonas florescence significantly decrease disease compared to infected control^52^. The findings are consistent with reports that *Ralstonia solanacearum* infections have a significant impact on crops; however, inoculation with Bacillus and pseudomonas species inhibits the growth of the pathogen and decrease disease severity in many infected crops^53^.The use of biocontrol agents can improve plant growth and control various diseases without harming the environment. By developing plant-microbial interactions in the rhizosphere, beneficial biocontrol agents produce biofilms and secondary compounds including fengycin, iturin, bacillomycin, and surfactin that lower the population of plant pathogens^54^. By producing extracellular enzymes as chitosanase, protease, glucanase, and cellulase, *Bacillus* species adhere to mycelial cell walls and deform hyphae^55^. Fengycin, iturin, pumilacidin, mixirin, and surfactin are examples of lipopeptides, which are antifungal peptides that work against harmful fungus in rhizospheres^56^. By influencing nematode behavior, including feeding and reproduction, many bacteria, especially *Bacillus*,* Pseudomonas*, and *Burkholderia spp*. are known to suppress nematodes in a variety of plants^57^. A root-knot nematode infestation was successfully managed by biological treatment using *Bacillus* isolates, according to previous studies^58^. Through enhanced defense-related enzyme activity, including polyphenol oxidase, peroxidase, and phenylalanine ammonia-lyase (PAL), as well as root exudates and modifications like amino acids and polysaccharides, Bacillus species have also been shown to promote induced systemic resistance in plants against a variety of pathogens^59^.

It has been observed that the biocontrol bacteria *Bacillus* and *Pseudomonas* spp. are efficient against a variety of phytopathogens in important crops. *Bacillus velezensis* isolates were found to contain many biosynthetic gene clusters that are involved in the manufacture of secondary metabolites^60^. The creation of novel medications may benefit from the utilization of these chemically diverse bioactive metabolites. In order to guarantee sustainable agriculture, new research on the production of bioactive substances, chemical compositions, intriguing bioactive gene clusters, and biological uses of *B. velezensis* and allied *Bacillus* species would be useful in controlling plant diseases^61^.

It has been observed that the biocontrol bacteria *Bacillus* and *Pseudomonas spp*. are efficient against a variety of phytopathogens in important crops. *Bacillus velezensis* isolates were found to contain many biosynthetic gene clusters that are involved in the manufacture of secondary metabolites^60^. The creation of novel medications may benefit from the utilization of these chemically diverse bioactive metabolites. In order to guarantee sustainable agriculture, new research on the production of bioactive substances, chemical compositions, intriguing bioactive gene clusters, and biological uses of B. *velezensis* and *Bacillus species* would be useful in controlling plant diseases^61^. In order to effectively control diverse crop diseases, a great deal of research should be done to create effective formulation technology and examine different natural products with the assessment of their antimicrobial activity both in vitro and in the field.

Disease reduction by *P. aeruginosa* and *B. thuringiensis* in colonization of plant roots may occur directly, through competition for space, nutrients and ecological niches or production of antimicrobial substances and indirectly, through Induction of Systemic Resistance (ISR)^52^. *Pseudomonas putida* and *pseudomonas florescence* may induce plant growth promotion by direct or indirect modes of action^53^. Directly by production of plant growth regulators and facilitation of the uptake of nutrients (nitrogen fixation, solubilization of phosphorus). The indirect by *pseudomonas putida* and *pseudomonas florescence* lessen or prevent the deleterious effects of plant pathogens on plants by production of inhibitory substances (antibiotics, antifungal metabolites, iron-chelating siderophores, cell wall-degrading enzymes and competition for sites on roots) or by increasing the natural resistance of the host (induced systemic resistance). In vivo results clearly confirm that application of *pseudomonas putida* and *pseudomonas florescence* as potential bio agents in controlling potato bacterial wilt under greenhouse condition. In this respect, the results of gene expression analysis revealed that potato plant developed a diversity of defense mechanisms against pathogenic fungi of the *Ralstonia solanacearum*, including transcriptional activation of pathogenesis-related (PR) genes. Through, systemic acquired resistance (SAR), which stimulates the expression of the *PR1*,* PR2*, and *PQ* genes and stops the spread of infection to healthy tissues, is mediated by the SA pathway, which is mostly activated by biotrophic pathogens [2]. In particular, expression of PR-1, PR-2 and PR-Q expression genes increased up to 16 folds, supporting that PR gene family play important roles in biotic stress response in plants. The results were agreement with those reported by Guo^62^, who reported that *R. solanacearum* wilt disease reduction and yield increase of potato plants after treatment by *Pseudomonas spp*. Also^52^ recorded 96% reduction of the potato bacterial wilt disease under greenhouse conditions using *Pseudomonas spp.*

In this investigation, biological control can be used on farms by integrating bio control agents into IPM plans. As a result, overall effectiveness is increased while reliance on chemically manufactured inputs is reduced. In the horticultural area, since many crops lack chemical synthetic PPPs that are legally allowed, this is very important. Furthermore, considering the short time between harvest and consumption, the problem of residues on harvested product is quite important^52^. Although chemical synthetic PPPs continue to be more cost-effective than the new alternatives and the process of changing the plant production system, these circumstances are driving the growth of alternative approaches. Biocontrol encourages biodiversity at the large-scale field level by supporting field management with a variety of habitats and ecosystems, creating an environment that supports the growth of beneficial microbes and native predators. By reducing pest pressure without only depending on chemical synthetic pesticides, this method helps preserve the equilibrium between pests and their natural enemies, which lowers the risk to human and animal health^62^. Biocontrol makes it possible to produce plants that meet quality standards like durability and resistance to secondary pests, which are impossible to achieve with conventional pesticides alone. By reducing plant diseases pressure without only depending on chemical synthetic fungicide, which lower the risk to human and animal health. By using bio control, it is possible to meet quality requirements in plant production, durability, and resistance to plant diseases.

## Conclusion

Potato plants are continuously challenged by brown rot, compromising food security. These pathogens can significantly reduce the potato productivity worldwide, with annual crop yield losses. But the use of synthetic chemicals on potato crops has detrimental effects on the environment and human health. Therefore, researchers worldwide have shifted their focus towards alternative eco-friendly strategies to prevent plant diseases. A bio control agent is a less toxic and safer method that reduces the severity of various crop diseases. They have evolved different mechanisms to counterattack brown rot infection, including the overexpression of pathogenesis-related (PR) proteins under treatment with biocontrol agents. Therefore, the objective of this investigation is the identification of the microbial community in brown rot and its function to suppress devastating plant diseases. Besides evolution, the bioefficacy of two biocontrol agents, either alone or in consortium, on plant growth promotion and activation of defense responses in potato against the brown rots diseases. The results of identification showed two separate bands belonging to Pseudomonas spp. with the accession numbers PQ466864 and PQ470140. While the results of the biocontrol microbe consortium not only induce defense systems in potatoes but also enhance plant growth promotion activities. Through potato tubers primed with the consortium and inoculated with pathogens exhibited a significant increase in total defense-related genes (PR-1, PR-2, and PR-Q) compared to the control plants challenged with *R. solanacearum*. In conclusion, this study demonstrates that brown rot enhances plant resistance against *R. solanacearum* infection through a biocontrol agent, which improves chances for resolving agricultural problems; it is possible to meet quality requirements in plant production, durability, and managing brown rot in potatoes in a sustainable way. Future biocontrol methods must be developed to guarantee effective crop disease management for sustainable agriculture.

## Data Availability

The raw data will be available on request. Correspondence and requests for materials should be addressed to H.S.O. The nucleotide sequence of the Pseudomonas spp, and P. putida isolate were deposited in Gene Bank with Accession No. PP930812, PQ466864, respectively but the nucleotide sequence of P. plecoglossicida isolate with Accession No PQ470140 will release through few days and this sequence will be available on request. Correspondence and requests for materials should be addressed to H.S.O.
